# Prognostic Factors and Survival Benefits of Antitumor Treatments for Advanced Non-Small Cell Lung Cancer Patients With Central Nervous System Metastasis With or Without Driver Genes: A Chinese Single-Center Cohort Study

**DOI:** 10.3389/fonc.2022.879554

**Published:** 2022-04-26

**Authors:** Xiaoxing Gao, Minjiang Chen, Xiaoyan Liu, Yuequan Shi, Hongge Liang, Qing Zhou, Jing Zhao, Ruili Pan, Wei Zhong, Yan Xu, Mengzhao Wang

**Affiliations:** Department of Respiratory and Critical Care Medicine, Peking Union Medical College Hospital, Chinese Academy of Medical Sciences and Peking Union Medical College, Beijing, China

**Keywords:** central nervous system metastasis, non-small-cell lung cancer, cohort study, prognostic factors, treatment outcome

## Abstract

**Background:**

The prognosis of non-small cell lung cancer (NSCLC) patients with central nervous system (CNS) metastasis is poor. The treatment for CNS metastasis could prolong the overall survival of NSCLC patients. We aimed to investigate the prognostic factors of Chinese NSCLC patients with CNS metastasis and the survival benefits of various treatments for CNS metastasis in NSCLC patients with or without driver genes.

**Methods:**

Based on the CAPTRA-Lung database, NSCLC patients with CNS metastasis admitted at the Peking Union Medical College Hospital between January 2010 and October 2018 were enrolled in the study. The prognostic factors were analyzed using univariate and multivariate Cox regression analyses.

**Results:**

Overall, 418 patients were enrolled in the study. A total of 206 patients (49.3%) had CNS metastasis with positive driver genes, while 97 patients (23.2%) had negative driver genes. The median survival time after CNS metastasis was 20.8 months. In the multivariable analysis, an Eastern Cooperative Oncology Group performance status of ≥2 (hazard ratio [HR]: 1.750, 95% confidence interval [CI]: 1.184-2.588, P=0.005), number of CNS metastases ≥5 (HR: 1.448, 95% CI: 1.084 -1.934, P=0.012), and CNS metastasis developed during treatment (HR: 1.619, 95% CI: 1.232-2.129, P=0.001) were independent risk factors for poor survival. Lung adenocarcinoma (HR: 0.490, 95% CI: 0.279-0.861, P=0.013) and driver gene positivity (HR: 0.464, 95% CI: 0.302-0.715, P=0.001) were independent predictors of prolonged survival. Radiotherapy for CNS metastasis showed a survival benefit in NSCLC patients in the entire groups (HR: 0.472, 95% CI: 0.360-0.619, P <0.001), and in patients with positive driver genes.

**Conclusion:**

Performance status, number of CNS metastases, timing of CNS metastasis, histological subtype, and driver gene status are prognostic factors for NSCLC patients with CNS metastasis. Furthermore, radiotherapy improved the survival in NSCLC patients with CNS metastasis.

## Introduction

Lung cancer is the leading cause of cancer-related deaths worldwide (1). Non-small cell lung cancer (NSCLC) accounts for 85% of all lung cancers. The central nervous system (CNS) is one of the most common sites of distant metastasis from advanced NSCLC (2). Due to the severe clinical symptoms and the serious effects on the quality of life of these patients, CNS metastasis serves as one of the main causes of death in patients with NSCLC, and is drawing increasing clinical and research attention in recent years. CNS metastasis includes brain metastasis (BM), leptomeningeal metastasis (LM), intramedullary spinal cord metastasis (ISCM), etc. (2, 3). BM is the most common type of CNS metastasis in NSCLC patients and is found in up to 40–50% of advanced NSCLC patients during their disease course. LM is defined as one of the most devastating events of advanced NSCLC, with a median overall survival (OS) of 3–11 months (4, 5). ISCM is a rare type of metastasis, with severe disabilities and poor prognosis (6), and should also be considered in clinical practice.

In NSCLC patients with CNS metastasis, the known disease-related prognostic factors include the number of BM, age, Karnofsky performance score (KPS), extracranial metastases (ECM), and driver gene status (7–9). Local and systemic treatments are used for managing NSCLC patients with CNS metastasis. The appropriate treatment for CNS metastasis could relieve neurological symptoms, improve quality of life, and prolong OS (2, 10). Owing to the development of small-molecule targeted drugs and immune checkpoint inhibitors (ICIs), as well as new surgical techniques and radiotherapy techniques, the systemic treatment and local treatment paradigms for CNS metastasis from NSCLC with or without driver genes are different. Therefore, careful evaluation of the specific metastatic sites and the severity of CNS metastasis as well as primary tumors is important for the treatment and prognosis of patients.

In order to investigate the prognostic factors of Chinese NSCLC patients with CNS metastasis and to evaluate the survival benefits of various treatments for CNS metastasis in NSCLC patients with or without driver genes, we summarized a single-center experience based on the CAPTRA-Lung database.

## Materials and Methods

### Patients

Patients with NSCLC complicated by CNS metastases were enrolled in this study. Patients (1) whose NSCLC was diagnosed by pathological or cytological examination; (2) whose CNS metastases were diagnosed by radiological imaging and/or confirmed by pathological examination; (3) with complete data on the diagnosis and treatment of NSCLC, especially those related to CNS metastasis, were included in the study. Meanwhile, patients (1) who were diagnosed with small cell lung cancer based on the results of pathological examination, (2) whose primary malignancy does not originate from the lungs, (3) who were not clearly diagnosed with CNS metastasis, and (4) with incomplete clinical information, were excluded. The keywords “NSCLC” and “central nervous metastatic cancer” were used to search the electronic medical record system from January 1st, 2010, to October 31st, 2018, in Peking Union Medical College Hospital. A total of 716 patients were retrieved from the electronic medical record system. Seventy-two patients did not meet the inclusion criteria, and 177 patients without detailed information were excluded. The data of 467 patients were collected using the CAPTRA-Lung database. Data integrity analysis, data confirmation, and supplementation were completed. Finally, 49 patients without complete CNS metastasis information were excluded, and 418 patients were enrolled in this study. This study was approved by the Ethics Committee of Peking Union Medical College Hospital.

### Methods

Patients’ medical records were reviewed, and the following information were collected: sex, age, past medical history, history of malignancies, family history of malignancies, diagnosis and staging of lung cancer, sites of metastasis, pathology-related information, driver gene mutation status, tumor treatment, and prognosis. The NSCLC driver gene mutation information was obtained from the medical records. The EGFR mutations were determined using ARMS or next-generation sequencing (NGS); ALK rearrangement was determined using immunohistochemistry, fluorescent *in situ* hybridization (FISH), or NGS method; the ROS-1 rearrangement was determined using FISH or NGS method; and the other genetic alterations were evaluated using the NGS method. Positive driver genes are defined as positive driver genes that can be treated by available targeted therapy, including EGFR mutation, ALK rearrangement, ROS-1 rearrangement, RET rearrangement, c-MET amplification or c-MET exon14 skipping, etc. Patients with positive driver genes were enrolled in positive driver genes group, but patients with KRAS mutations were not included in the positive driver genes group as there was no specific targeted therapy for KRAS mutation at the time of the study. Patients with negative EGFR mutation and negative ALK rearrangement, and without identified positive driver genes, were included in the negative driver genes group. Driver genes status unknown patients were included in the driver genes status unknown group.

Information related to patients with CNS metastasis was collected, including symptoms of CNS metastasis, time of first CNS metastasis diagnosis, site of CNS metastasis, number of CNS metastatic foci, and treatment for CNS metastasis. The time of the first diagnosis of CNS metastasis was defined as the date when CNS metastasis was initially detected by enhanced magnetic resonance imaging (MRI) or computed tomography (CT) imaging. If the patient had LM without BM, the time of the first diagnosis was defined as the data when LM was initially identified by enhanced MRI or cerebrospinal fluid cytology. CNS symptoms were collected at the time of the first diagnosis of CNS metastases. The CNS metastasis type is determined according to the CNS metastasis sites involved (BM, LM, ISCM, etc.) that occurred throughout the course of the disease. The number of CNS metastatic lesions was calculated according to the CNS metastatic lesions shown on imaging at the time of the first diagnosis of CNS metastasis. Since LM was diffusely spread in the subarachnoid space, all patients with LM were documented as having ≥5 lesions. Systemic antitumor therapy for CNS metastasis includes targeted therapy, chemotherapy, antiangiogenesis therapy, and immunotherapy. Patients with EGFR-sensitive mutations received first-line targeted therapy, including gefitinib, erlotinib, and icotinib, while some patients were received osimertinib as a second-line treatment. Patients with ALK rearrangement received targeted therapy, including crizotinib, ceritinib, and alectinib. The antiangiogenesis therapy consisted mainly of bevacizumab. CNS metastasis-related local antitumor therapy includes surgery for BM and radiotherapy. Whole brain radiotherapy (WBRT) and stereotactic radiotherapy (SRT) were all recorded as CNS radiotherapy.

### Statistical Analysis

Patient registration and standardized data collection were performed using the CAPTRA-Lung database. Data integrity analysis of the enrolled patients was performed, and the missing data were supplemented by careful follow-up, with the last follow-up conducted on September 20th, 2019. Finally, complete data were exported from the CAPTRA-Lung database for analysis.

OS is defined as the time from the first diagnosis of CNS metastasis until death or the last follow-up. If the patient was alive or lost to follow-up, OS was calculated based on the time of the patient’s last visit and was indicated as censored data. Categorical variables were expressed as numbers and/or percentages. The chi-square test was used to compare the differences between the groups. Continuous variables were expressed as average ± standard error. Survival data were analyzed using the Kaplan–Meier method. The OS of patients from different subgroups was compared using the log-rank test. Multivariate Cox regression analysis was used to analyze the factors influencing the prognosis. SPSS 17.0 statistical software (SPSS Inc., Chicago, USA) was used to perform all statistical analyses and GraphPad Prism (GraphPad Software, La Jolla, CA, USA) was used for statistical graphic drafting. A P value ≤0.05 was considered statistically significant.

## Results

### Demographic Information

A total of 418 patients were enrolled in this study; their baseline information are listed in **Table 1**. Fifty-five patients were initially diagnosed with stage I–III NSCLC, of whom 53 underwent surgery and 2 had concurrent radiochemotherapy. A total of 363 patients initially diagnosed with stage IIIB or IV NSCLC were not candidates for resection or were not suitable for radiotherapy. The histopathological distribution was as follows: 362 patients had lung adenocarcinoma (LUAD) (86.6%), 25 had squamous cell lung carcinoma (LUSC) (6.0%), and 31 (7.4%) had other types of malignancy. Of all patients with CNS metastasis, 206 (49.3%) harbored a positive driver gene. EGFR mutations, ALK rearrangement mutations, and ROS-1 rearrange mutations were detected in 179 patients (42.8%), 27 patients (6.5%), and 1 patient, respectively. Among them, one patient had a combination of EGFR and ALK fusion gene mutations, while another patient had RET rearrangement combined with EGFR mutation. A total of 97 patients (23.2%) had negative driver genes. The remaining 115 patients (27.5%) had unknown driver gene status.

**Table 1:** Clinical features of NSCLC patients with CNS metastases.

Characteristic	
Age	58.8 ± 11.6
Gender (M vs.F)	221 (52.9) vs.197 (47.1)
Smoking status (no vs. yes vs. unknown)	233 (55.7) vs. 168 (40.2) vs.17 (4.1)
History of malignancy, n (%)	67 (16.0)
Family history of malignancies, n (%)	102 (24.4)
Neurological symptoms at first diagnosis, n (%)	
No	222 (53.1)
Yes	196 (46.9)
ECOG, n (%)	
0–1	371 (88.8)
≥2	47 (11.2)
Histology subtype, n (%)	
LUSD	25 (6.0)
LUAD	362 (86.6)
Others	31 (7.4)
Driver genes status, n (%)	
EGFR positive, n (%)	179 (42.8)
EGFR Ex18	5 (1.2)
EGFR Ex19	68 (16.3)
EGFR Ex20	5 (1.2)
EGFR Ex21	84 (20.1)
EGFR Unknow	17 (4.1)
ALK rearrangement, n (%)	27 (6.5)
ROS-1 rearrangement, n (%)	1 (0.2)
RET rearrangement, n (%)	1 (0.2)
Driver gene negative, n (%)	97 (23.2)
Driver gene unknown, n (%)	115 (27.5)
Site of metastasis, n (%)	
BM	394 (94.3)
LM	67 (16.0)
BM and LM	43 (10.3)
BM and ISCM	1 (0.2)
Number of CNS metastasis, n (%)	
<5	190 (45.5)
≥5	200 (47.8)
Others/unknown	28 (6.7)
Timing of CNS metastasis occurrence, n (%)	
Treatment naive	276 (66.0)
During treatment follow-up	142 (34.0)

Values are mean ± SD, n (%).

ALK, anaplastic lymphoma kinase; BM, brain metastasis; CNS, central nervous system; ECOG, Eastern Cooperative Oncology Group; EGFR, epidermal growth factor receptor; F, female; ISCM, intramedullary spinal cord metastasis; LM, leptomeningeal metastasis; LUAD, lung adenocarcinoma; LUSC, squamous cell lung carcinoma; M, male; NSCLC, non-small cell lung cancer.

### CNS Metastasis and Treatment

A total of 276 (66.0%) patients had CNS metastasis at the time of lung cancer diagnosis. A total of 142 patients (34.0%) were found to have CNS metastasis at treatment follow-up. The median time from the initial diagnosis of lung cancer to the time of CNS metastasis detection was 12.3 months (range: 1.2 months–13.5 years). A total of 222 (53.1%) patients were asymptomatic at time of CNS metastasis detection by routine imaging examination. A total of 196 (46.9%) patients were found to have CNS metastasis due to the presence of neurological symptoms. The most common symptoms included headache (95/196, 48.5%), dizziness (70/196, 35.7%), muscle weakness (38/196, 19.4%), nausea (38/196, 19.4%), projectile vomiting (34/196, 17.3%), unstable walking (25/196, 12.8%), blurred vision (19/196, 9.7%), and sensory disorder (16/196, 8.1%).

Moreover, 394 (94.3%) and 67 (16.0%) patients had BM and LM, respectively. Forty-three (10.3%) patients had concurrent BM and LM. One patient (0.2%) had concurrent BM and ISCM. Most patients (79.1%,53/67) with LM developed definite neurological symptoms, including headache (56.7%, 38/67), dizziness (35.8%, 24/67), ejective vomiting (34.3%, 23/67), blurred vision (14.9%, 10/67), etc.

A total of 262 (62.7%) patients received targeted therapy, 232 (55.5%) received chemotherapy, 4 (1.0%) received immunotherapy, and 31 (7.4%) received antitumor angiogenesis therapy. Local treatment was performed in 197 patients (47.1%), including 44 patients (10.5%) who underwent neurosurgery (14 of whom received CNS radiotherapy) and 167 patients (40.0%) who received CNS radiotherapy. Patients who underwent neurosurgery had more neurological symptoms than those treated with radiotherapy (38/44 [86.4%] vs. 95/167 [56.9%], P<0.001). However, the number of CNS metastases less than 5 in the neurosurgery group was 36 patients (36/44, 81.8%), while that in the CNS radiotherapy group was 58 patients (58/167, 34.7%), with significant difference (P<0.001).

### Driver Genes Status and Clinical Characteristics of CNS Metastasis

We further analyzed the clinical characteristics of the three groups of patients with positive driver genes, negative driver genes, and driver genes status unknown (**Table 2**). Patients with positive driver genes were more likely to have LM (positive driver genes: 24.3% vs. negative driver genes: 9.3%, P=0.002) and had more CNS lesions (the number of CNS metastases ≥5: positive driver gene 58.3% vs. negative driver gene 42.3%, P<0.001). Moreover, patients with positive driver genes had a higher percentage of developing metastasis during treatment (positive driver gene: 38.3% vs. negative driver gene: 20.6%; P=0.002). For patients with different driver gene status, the proportion of patients receiving local treatment, including brain radiation or brain surgery, was comparable. From the systemic treatment aspect, almost all the patients with positive driver genes received targeted therapy (positive driver gene: 98.5% vs. negative driver gene: 24.7%, P<0.001); a lesser proportion of patients with positive driver genes received chemotherapy compared with that of patients with negative driver genes (positive driver gene 44.2% vs. negative driver gene 75.3%, P<0.001).

**Table 2:** Clinical characteristics of NSCLC patients with positive driver genes, negative driver genes, and unknown driver genes status.

	Positive driver genes group (N=206)	Negative driver gene group (N=97)	Driver genes status unknown (N=115)	*p*-value#	*p*-value*
Age	57.5 ± 12.3	59.7 ± 10.6	60.5 ± 10.9	0.060	0.124
Gender (M:F)	81(39.3):125 (60.7)	71 (73.2): 26 (26.8)	69 (60.0): 46 (40.0)	<0.001	<0.001
Smoking status				<0.001	<0.001
Never smoker	140 (68.0)	33 (34.0)	60 (52.2)		
Smoker	55 (26.7)	62 (63.9)	51 (44.3)		
Unknown	11 (5.3)	2 (2.1)	4 (3.5)		
Neurological symptoms					
No	113 (54.9)	54 (55.7)	60 (52.2)	0.857	0.894
Yes	93 (45.1)	43 (44.3)	55 (47.8)		
ECOG					
0–1	184 (89.3)	86 (88.7)	101 (87.8)	0.920	0.863
≥2	22 (10.7)	11 (11.3)	14 (12.2)		
Histology subtype					
LUSD	0	7 (7.2)	18 (15.7)	<0.001	<0.001
LUAD	199 (96.6)	80 (82.5)	83 (72.2)		
Others	7 (3.4)	10 (10.3)	14 (12.2)		
CNS metastasis type					
No LM group	156 (75.7)	88 (90.7)	107 (93.0)	<0.001	0.0021
LM group	50 (24.3)	9 (9.3)	8 (7.0)		
Number of CNS metastasis					
<5	78 (37.9)	46 (47.4)	66 (57.4)	0.010	<0.001
≥5	120 (58.3)	41 (42.3)	39 (33.9)		
Unknown	8 (3.9)	10 (10.3)	10 (8.7)		
Timing of Metastasis					
Treatment naïve	127 (61.7)	77 (79.4)	72 (62.6)	0.007	0.002
During treatment	79 (38.3)	20 (20.6)	43 (37.4)		
Treatment					
Neurosurgery	13 (6.3)	11 (11.3)	20 (17.4)	0.008	0.130
Radiotherapy	93 (45.1)	36 (37.1)	38 (33.0)	0.085	0.187
Chemotherapy	91 (44.2)	73 (75.3)	68 (59.1)	<0.001	<0.001
Target therapy	203 (98.5)	24 (24.7)	35 (30.4)	<0.001	<0.001
Immunotherapy	0	3 (3.1)	1 (0.9)	-	-
Antiangiogenesis therapy	20 (9.7)	10 (10.3)	1 (0.9)	<0.001	0.870

Values are mean ± SD, n (%).

^#^Among three groups.

*Positive driver gene group vs. negative driver gene group.

BM, brain metastasis; CNS, central nervous system; ECOG, Eastern Cooperative Oncology Group; F, female; LM, leptomeningeal metastasis; LUAD, lung adenocarcinoma; LUSC, squamous cell lung carcinoma; M, male; NSCLC, non-small cell lung cancer.

### Survival Outcomes

**Figure 1:**
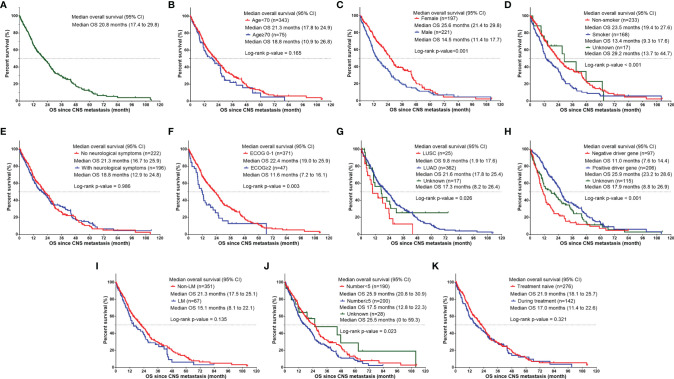
Kaplan–Meier curve showing OS since CNS metastasis developed based on different factors for NSCLC patients. **(A)** The median OS of NSCLC patients with CNS metastasis. The median OS since CNS metastasis developed **(B)** in NSCLC patients of age < 70 group vs. age≥70 group, **(C)** in female patients vs. male patients, **(D)** in NSCLC patients with different smoking status (non-smoker, smoker, and unknown), **(E)** in NSCLC patients without neurological symptoms vs. with neurological symptoms, **(F)** in NSCLC patients of ECOG 0-1 vs. ECOG ≥ 2, **(G)** in NSCLC patients of different histology subtype (LUSC, LUAD and unknown), **(H)** in NSCLC patients with different driver gene status (negative driver gene, positive driver gene, and unknown), **(I)** in NSCLC patients with non-LM vs. with LM, **(J)** in NSCLC patients with CNS metastasis number <5 vs. CNS metastasis number≥5 vs. unknown, and **(K)** in NSCLC patients with CNS metastasis developed during treatment vs. treatment naïve. CNS, central nervous system; ECOG, Eastern Cooperative Oncology Group; LM, leptomeningeal metastasis; LUAD, lung adenocarcinoma; LUSC, squamous cell lung carcinoma; NSCLC, non-small cell lung cancer; OS, overall survival.

**Figure 2:**
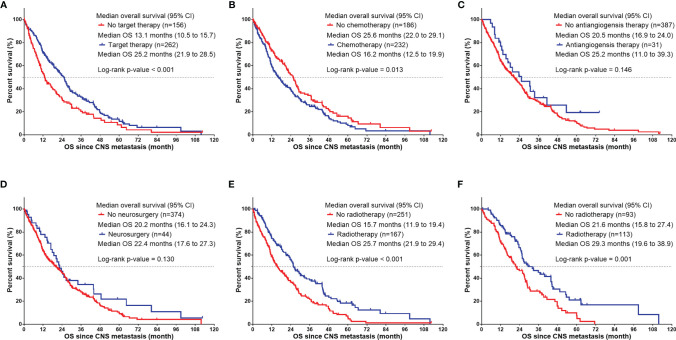
Kaplan-Meier curve illustrating OS since CNS metastasis in NSCLC patients with various treatments: **(A)** with or without target therapy, **(B)** with or without chemotherapy, **(C)** with or without antiangiogensis therapy, **(D)** with or without neurosurgery, **(E)** with or without radiotherapy in all NSCLC patients with CNS metastasis, and **(F)** with or without radiotherapy in positive driver genes NSCLC patients with CNS metastasis. OS, overall survival; CNS, central nervous system; NSCLC, non-small cell lung cancer.

**Figure 3:**
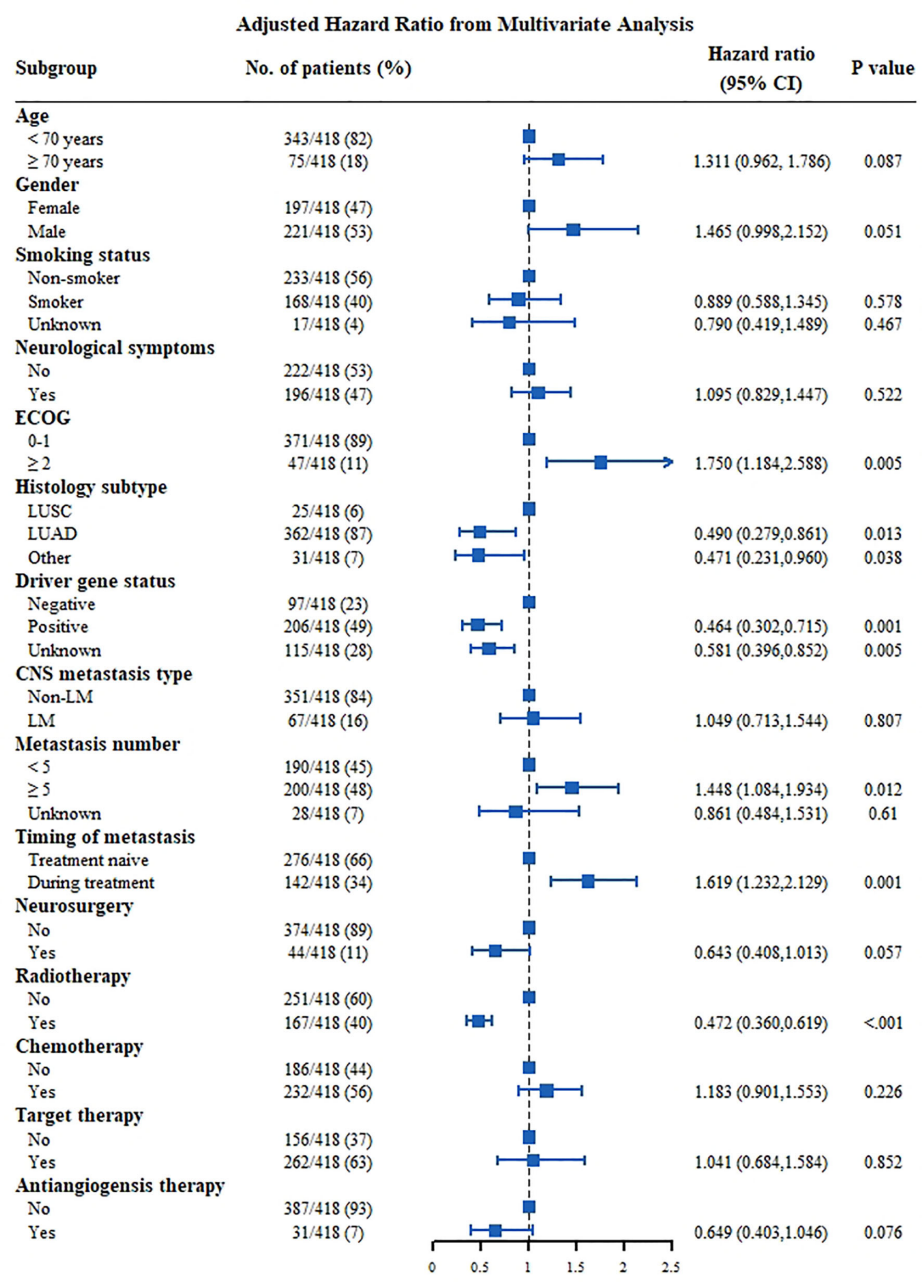
Multivariate analysis Cox proportional hazards for OS in NSCLC patients with CNS metastasis. CNS, central nervous system; ECOG, Eastern Cooperative Oncology Group; LM, leptomeningeal metastasis; LUAD, lung adenocarcinoma; LUSC, squamous cell lung carcinoma.

The median OS of the whole group was 20.8 months (**Figure 1A**). Univariate analysis was performed based on the patient population (**Figures 1B–K**). Man (women: 25.6 months vs. men: 14.5 months, p<0.001, **Figure 1C**), smokers (non-smoker: 23.5 months vs. smoker: 13.4 months vs. unknown: 29.2 months, P<0.001, **Figure 1D**), those with ECOG score ≥2 (ECOG score 0–1: 22.4 months vs. ECOG≥2: 11.6 months, P=0.003, **Figure 1F**), and those with ≥5 CNS metastases (<5 CNS metastases: 25.9 months vs. ≥5 CNS metastases: 17.5 months vs. unknown: 25.5 months, P=0.023, **Figure 1J**) had a shorter OS. LUAD patients had longer survival (LUSC: 9.8 months vs. LUAD: 21.6 months vs. other subtype 17.3 months P=0.026, **Figure 1G**). Patients with positive driver genes had longer OS (negative driver gene: 11.0 months vs. positive driver gene: 25.9 months vs. driver genes status unknown: 17.9months, P<0.001, **Figure 1H**). In terms of treatment (**Figures 2A–F**), patients who received targeted therapy had longer OS (no targeted therapy: 13.1 months vs. targeted therapy: 25.2 months, P<0.001, **Figure 2A**), and patients receiving radiotherapy had longer OS (no radiotherapy: 15.7 months vs radiotherapy: 25.7 months, P<0.001, **Figure 2E**). However, the OS of patients with chemotherapy was poor (no chemotherapy: 25.6 months vs. chemotherapy: 16.2 months, P=0.013, **Figure 2B**). Patients receiving antiangiogenesis therapy showed a tendency to have prolonged OS (no antiangiogenesis therapy: 20.5 month vs. antiangiogenesis therapy: 25.2months, P=0.146, **Figure 2C**), without significant difference. Multivariate analysis (**Figure 3**) showed that an EOCG score ≥2 (hazard ratio [HR]: 1.75, 95% confidence interval [CI]: 1.184–2.588, P=0.005), number of CNS metastases ≥5 (HR: 1.448, 95% CI: 1.084 -1.934, P=0.012), and CNS metastasis that developed during treatment (HR: 1.619, 95% CI: 1.232–2.129, P=0.001) were independent risk factors for poor prognosis. Patients with LUAD (HR: 0.490, 95% CI: 0.279–0.861, P=0.013) and driver gene positivity (HR: 0.464, 95% CI: 0.302–0.715, P=0.001) were independent predictors of prolonged survival. Radiotherapy for CNS metastasis showed a survival benefit in patients with NSCLC (HR: 0.472, 95% CI: 0.360–0.619, P<0.001).

**Figure 4:**
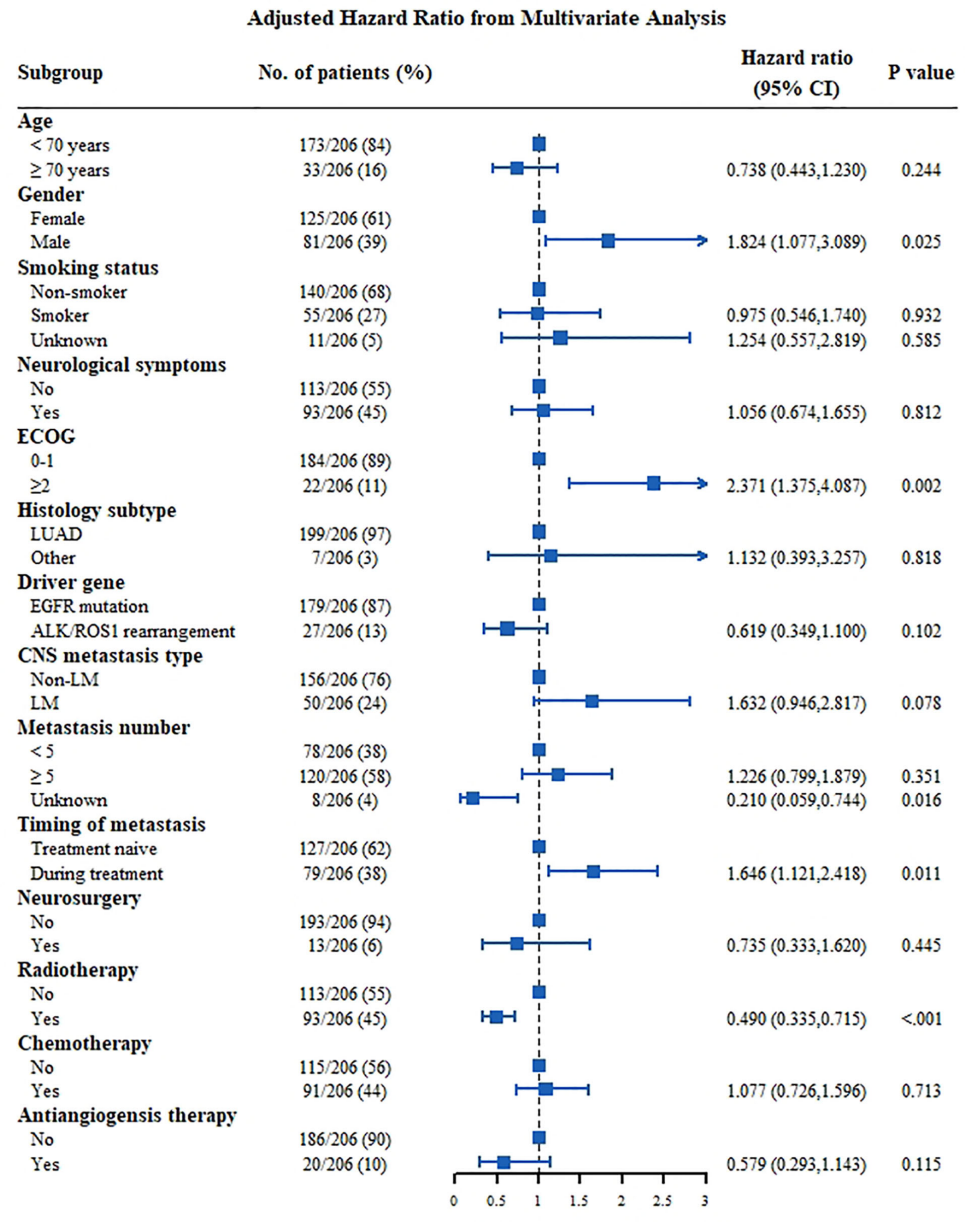
Multivariate analysis Cox proportional hazards for OS in NSCLC patients with CNS metastasis of positive driver genes group. ALK, anaplastic lymphoma kinase; CNS, central nervous system; ECOG, Eastern Cooperative Oncology Group; EGFR, epidermal growth factor receptor; LM, leptomeningeal metastasis; LUAD, lung adenocarcinoma; LUSC, squamous cell lung carcinoma.

For patients with positive driver genes (**Figure 4**), male patients (HR: 1.824, 95% CI: 1.077–3.089, P=0.025), patients with an ECOG score ≥2 (HR: 2.371, 95% CI: 1.375–4.087, P=0.002), and patients with CNS metastasis developed during follow-up had a poor prognosis (HR: 1.646, 95% CI: 1.121–2.418, P=0.011). Meanwhile, patients who received radiotherapy had a good prognosis (HR: 0.490, 95% CI: 0.335–0.715, P<0.001).

**Figure 5:**
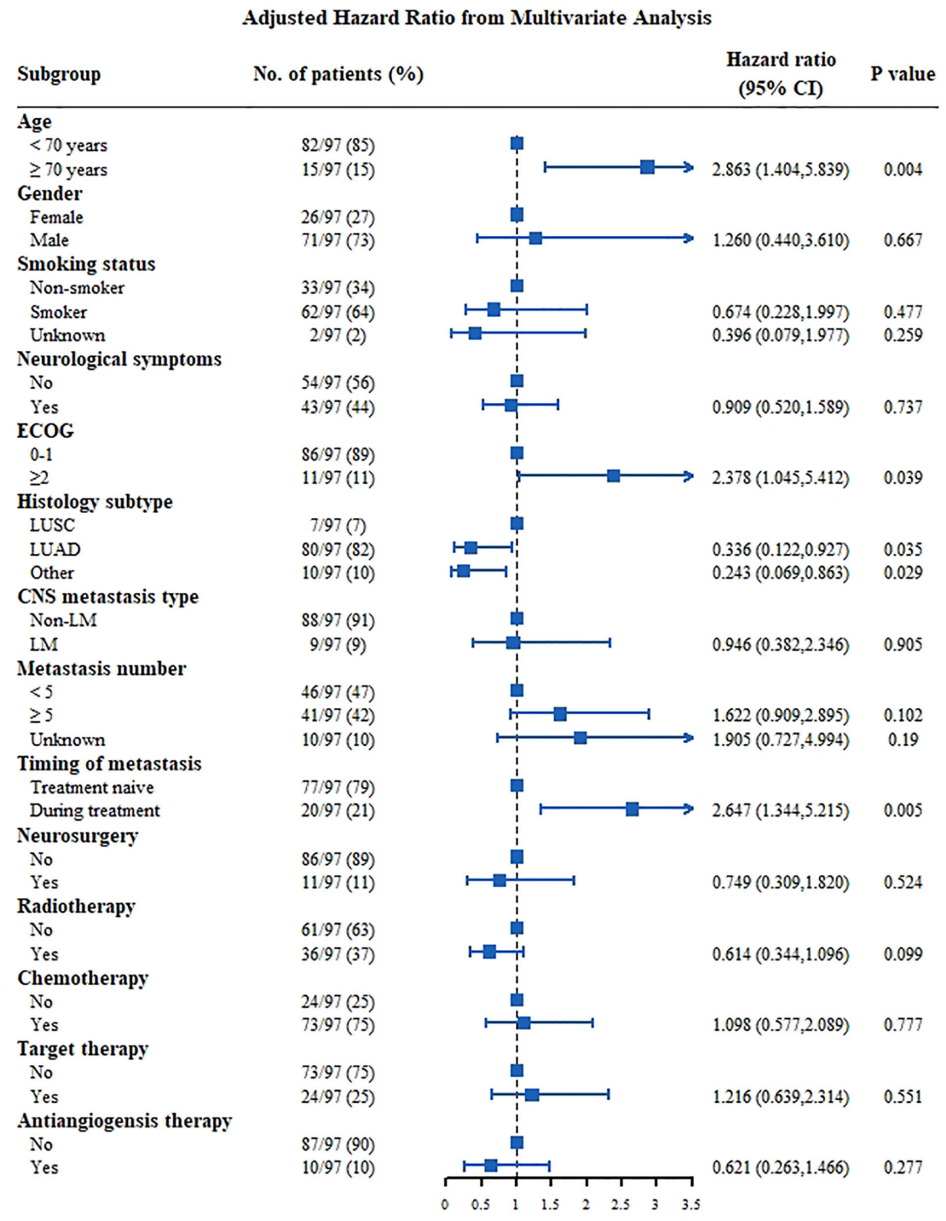
Multivariate analysis Cox proportional hazards for OS in NSCLC patients with CNS metastasis of negative driver genes group. CNS, central nervous system; ECOG, Eastern Cooperative Oncology Group; LM, leptomeningeal metastasis; LUAD, lung adenocarcinoma; LUSC, squamous cell lung carcinoma.

Results of the multivariate analysis showed that for patients with negative driver genes (**Figure 5**), those aged ≥70 years (HR: 2.863, 95% CI: 1.404-5.839], P=0.004) and with an ECOG score ≥2 (HR: 2.378, 95% CI: 1.045-5.412, P=0.039) had a poor prognosis, and patients with CNS metastasis during treatment (HR: 2.647, 95% CI: 1.344–5.215] P=0.005) had a poor prognosis as well. Patients with LUAD (HR: 0.366, 95% CI: 0.122–0.927, P=0.035) were independent predictors of prolonged survival. The effect of the treatment appeared to be insignificant.

## Discussion

This study summarized and analyzed the clinical characteristics of patients with CNS metastasis of NSCLC in a Chinese single-center hospital. Our study indicates that: (1) 49.3% of patients with NSCLC with positive driver genes were more likely to develop multiple CNS metastases and LM; (2) EOCG score ≥2, metastasis ≥5, and CNS metastasis that developed during treatment were independent risk factors for poor prognosis, while lung adenocarcinoma and positive driver genes were independent predictors of long-term survival; and (3) patients who received radiotherapy had longer survival.

Many studies have analyzed the prognostic factors of CNS metastasis in patients with NSCLC. The Diagnosis-Specific Graded Prognostic Assessment (DS-GPA) score (7) was used to assess the disease-specific grading prognosis of tumor patients with BM, and KPS, age, ECM, and number of BM are the key prognostic factors included in the lung cancer GPA scoring criteria. EGFR or ALK gene status is included in the amendment of lung cancer score, lung-molGPA (8, 11). For patients with LM, in Yin et al.’s study (9), KPS, ECM, and gene status were proposed as prognostic factors in lung cancer patients with LM. Steindl et al. (12) believed that the neurological symptom burden of BM was negatively correlated with the prognosis of NSCLC. However, most models do not include CNS symptoms. Moreover, no significant prognostic correlation was observed among all symptoms evaluated in our study. In this study, performance status and number of BM were still considered as significant prognostic factors. In addition, the occurrence of CNS metastasis during treatment was included in the prognostic criteria. Patients who developed CNS metastasis during treatment, whether in the whole population or in the group with positive or negative driver genes, had a poor prognosis. Due to the presence of a blood-brain barrier (BBB), the CNS is considered as the tumor cell havens for drug resistance during antitumor treatment, while the subarachnoid space is considered as the ultimate shelter of tumor cells. For patients who developed CNS metastasis during treatment, drug resistance, few treatment options, poor performance status, and poor treatment tolerability result in poor prognosis.

Driver gene status is an important factor affecting the prognosis of NSCLC patients with CNS metastasis. NSCLC patients with positive or negative driver genes have different characteristics in terms of CNS metastasis. Patients with positive driver genes were more likely to develop CNS metastases. Patients with advanced EGFR mutations were significantly more likely to have BM than those with wild-type EGFR (70.3% vs. 38.1%, P=0.002) (13), and patients with EGFR mutations were more likely to have LM (9.4vs. 1.7%, P <0. 001) (4). In addition, patients with ALK rearrangement and RET rearrangement are at risk of developing BM (14). This study showed that patients with driver genes were at higher risk of developing more CNS metastatic lesions and LM. Meanwhile, in this study, patients with positive driver genes were more likely to develop CNS metastasis during treatment. Benefitting from the target therapy, NSCLC patients with positive driver genes have a long duration of response, and the CNS can become an independent site for progression of drug resistance. Therefore, BM occurring during treatment is often observed, and even LM occurs during treatment. For NSCLC patients with negative driver genes, the probability of NSCLC CNS metastasis during treatment is low due to the poor systemic treatment effects and short survival periods. The prognostic factors are also different in patients with NSCLC CNS metastasis, with or without driver genes. In the study by Yu et al. (15), age, KPS, type of EGFR mutation, brain metastases of >3, and presence of extracranial metastases were prognostic factors for BM in EGFR-mutated NSCLC. For EGFR/ALK-negative patients (16), the number of BM and KPS scores influences the prognosis of patients with wild-type NSCLC. In this study, for NSCLC patients with positive driver genes, male patients, patients with poor ECOG scores, and patients with metastasis during treatment had a poor prognosis. For CNS patients with negative driver genes, older age, poor ECOG scores, and CNS metastasis that developed during treatment were predictors of poor prognosis; meanwhile, LUAD patients had a good prognosis.

The traditional treatment of NSCLC with CNS metastasis involves local and systemic treatment (10). Radiotherapy and neurosurgery are common local treatment approaches (10). For patients with marked neurological mass effect or isolated BM, neurosurgery allows tumor tissue sampling, preserves the neurological function, and improves the patient outcomes. In addition, ventricular peritoneal (VP) shunt was also beneficial to the management of patients with LM (17). Radiotherapy plays an important role in managing NSCLC patients with CNS metastasis, especially stereotactic radiosurgery (SRS), the currently recommended radiation strategy that is regarded as the most accurate method with minimal side effects, helping systems control and prolonging patients’ OS (10). In this study, radiotherapy significantly prolonged the survival of the entire group of patients with CNS metastasis, while neurosurgery tends to prolong the patient’s survival.

Systematic antitumor treatment is also an important strategy in the treatment of NSCLC with CNS metastasis (18). For patients with positive and negative driver genes, the effects of systemic therapy and the combination of systemic therapy and local therapy greatly differ. Small molecular targeting drugs have a potent ability to cross the BBB and significantly improve the CNS-related symptoms and survival in NSCLC patients with driver genes. Benefitting from the efficient research and development of drugs with high BBB penetration targeting NSCLC-related driver genes, NSCLC patients with positive driver genes who developed CNS metastases, including BM and LMs, received effective treatment (19). Furthermore, targeted therapy combined with SRS and/or WBRT was reported to prolong the patient’s OS in several studies (20, 21). The timing of radiotherapy for patients with positive driver genes remains controversial. Several studies (22) believe that SRS combined with targeted therapy should be administered early, which can significantly prolong the prognosis of patients. However, another study by our team (23) and Chen et al. (24) showed that, as long as radiotherapy is administered during the course of targeted therapy, the patients’ OS can be prolonged. The results of this study showed that radiotherapy with target therapy can improve the patient’s outcomes.

For those without driver genes, cytotoxic drugs are less effective in patients with CNS metastasis because of poor BBB penetration. However, previous studies have shown that pemetrexed combined with platinum-based therapy can prolong the survival in patients with a negative driver gene or unknown driver gene status (24). In addition, ICIs have brought about significant survival benefits for NSCLC patients without driver genes in recent years. Among these, the population of NSCLC patients with BM is also reported to benefit from ICI treatment (25, 26). In clinical trials of various stages of NSCLC, the intracerebral objective remission rate of ICIs was similar to the extracranial objective remission rate and had a lasting effect (27, 28). The combination of ICI with radiotherapy for BM has a good safety tolerance, and both local control rate and overall survival of patients are improved, compared with radiotherapy alone (29). Longer progression-free survival was also achieved with the addition of radiotherapy to ICIs (30, 31). Due to the research period of this study, immunotherapy has not been widely used in the Chinese population, and only a few patients were included. In this study, no treatment showed outcomes improvement in NSCLC patients with negative driver genes, demonstrating an urgent unmet clinical need.

In this study, the OS of patients treated with antiangiogenesis therapy was slightly prolonged, but there was no clear statistical difference due to the small sample size. The application of antiangiogenesis therapy for BM has some effect for asymptomatic brain metastases when combined with other treatments. Antiangiogenesis treatment can reduce BM-related brain edema, leading to improvement in neurological symptoms. Bevacizumab has a good symptom improvement effect for pseudo-progression or radioactive brain necrosis caused by radiotherapy. Current small-molecule antivascular agents, such as anlotinib, have been shown to significantly improve the BM-related edema or edema secondary to SRS (32).

This study has some limitations. (1) New treatment strategies that have already been developed, such as immunotherapy, were not included in this study, because of research period. (2) Based on the evaluation of the overall treatment mode, specific medication and radiotherapy modes were not further detailed. (3) The parameters of metastasis during treatment were included, but extracranial metastasis was not evaluated. (4) This single-center study was retrospective performed in real word, which requires further verification through a multi-center study.

In conclusion, by summarizing the characteristics, treatment, and prognosis of patients with single-center metastatic disease, ECOG score ≥2, metastasis number ≥5, and the occurrence of CNS metastasis during treatment were independent risk factors for poor prognosis, while LUAD and positive driver genes were independent predictors of long survival. Patients in the entire group, or in the driver gene-positive group, who received radiotherapy, had longer survival.

## Data Availability Statement

The original contributions presented in the study are included in the article/supplementary material. Further inquiries can be directed to the corresponding authors.

## Ethics Statement

The studies involving human participants were reviewed and approved by Ethics Committee of Peking Union Medical College Hospital. The patients/participants provided their written informed consent to participate in this study.

## Author Contributions

Conceptualization: MW and YX. Funding acquisition: YX. Methodology: YX. Resources: XG, MC, XL, YS, HL, QZ, JZ, RP, WZ, YX, and MW. Roles/Writing original draft: XG, MC, and YX. Validation: MW and YX. All authors contributed to the article and approved the submitted version.

## Funding

This study was supported by the Youth Program of the National Natural Science Foundation of China (to YX) (grant number: 82003309) and by CAMS Innovation Fund for Medical Sciences (CIFMS) (to YX) (grant number: 2021-I2M-C&T-B-014).

## Conflict of Interest

The authors declare that the research was conducted in the absence of any commercial or financial relationships that could be construed as a potential conflict of interest.

## Publisher’s Note

All claims expressed in this article are solely those of the authors and do not necessarily represent those of their affiliated organizations, or those of the publisher, the editors and the reviewers. Any product that may be evaluated in this article, or claim that may be made by its manufacturer, is not guaranteed or endorsed by the publisher.

## Abbreviations

NSCLC, non-small cell lung cancer; CNS, central nervous system; ECOG, Eastern Cooperative Oncology Group; HR, hazard ratio; CI, confidence interval; BM, brain metastasis; LM, leptomeningeal metastasis; ISCM, intramedullary spinal cord metastasis; OS, overall survival; KPS, Karnofsky performance score; ECM, extracranial metastases; ICIs, immune checkpoint inhibitors; EGFR, epidermal growth factor receptor; ALK, anaplastic lymphoma kinase; NGS, next-generation sequencing; FISH, fluorescent *in situ* hybridization; MRI, magnetic resonance imaging; CT, computed tomography; LUAD, lung adenocarcinoma; LUSC, squamous cell lung carcinoma; DS-GPA score, Diagnosis-Specific Graded Prognostic Assessment score; BBB, blood-brain barrier; VP shunt, ventricular peritoneal shunt; SRS, Stereotactic radiosurgery; WBRT, whole brain radiation therapy.
